# Possession Zone as a Performance Indicator in Football. The Game of the Best Teams

**DOI:** 10.3389/fpsyg.2017.01176

**Published:** 2017-07-14

**Authors:** Claudio A. Casal, Rubén Maneiro, Toni Ardá, Francisco J. Marí, José L. Losada

**Affiliations:** ^1^Department of Science of Physical Activity and Sport, Catholic University of Valencia “San Vte Mártir” Valencia, Spain; ^2^Department of Science of Physical Activity and Sport, Pontifical University of Salamanca Salamanca, Spain; ^3^Department of Physical and Sport Education, University of A Coruña A Coruña, Spain; ^4^Department of Methodology of Behavioral Sciences, University of Barcelona Barcelona, Spain

**Keywords:** observational methodology, football, offensive phase, possession ball, performance indicators

## Abstract

Possession time in football has been widely discussed in research but few studies have analyzed the importance of the field area in which possession occurs. The objective of this study was to identify the existence of significant differences in the field zone of ball possession between successful and unsuccessful teams and to acknowledge if the match status modulates the possession model. To this end, 2,284 attacks were analyzed corresponding to the matches in the final phase of the UEFA Euro 2016 France, recording possession time and field zone in which possession occurred. Video recordings of matches were analyzed and coded post-event using notational analysis. We have found that successful offensive game patterns are different from unsuccessful ones. Specifically, field zone in which major possession occurs changes significantly between successful and unsuccessful teams (*x*^2^ = 15.72, *p* < 0.05) and through Welch’s T significant differences were detected in possession time between successful and unsuccessful teams (*H* = 24.289, *p* < 0.001). The former are characterized by longer possession times, preferably in the middle offensive zone, on the other hand, unsuccessful teams have shorter possession times and preferably on the middle defensive zone. Logistic regression also allowed us to identify that greater possession in the middle offensive zone is a good indicator of success in the offensive game, allowing us to predict a greater chance of victory in the match. Specifically, every time the teams achieve possession in the middle offensive zone, the chance of winning the match will increase 1.72 times and, the probability of winning the match making longer possessions in the middle offensive zone is 44.25%. Applying the Kruskal–Wallis test we have also been able to verify how match status modulates the teams possession time, specifically, when teams are winning they have longer possessions *x*^2^ = 92.628, *p* = 0.011. Results obtained are expected to help gain more knowledge about successful offensive game models, as well as performance factors of the offensive phase, which will allow teams to optimize their training process and performance during the match.

## Introduction

In sports games and specifically in football, encounter analysis through systematic observation is an effective and objective instrument to collect information and identify the most relevant events that occur in them, as revealed by Carling et al. (2009) when affirming that match analysis has taken a transcendental role in sports.

In many cases observation is the only way to study a phenomenon without distorting it, watching it as it occurs in game context since, according to Anguera (1993), it is a particular strategy of the scientific method that proposes the quantification of spontaneous behavior that occurs in unprepared situations, implying that to achieve results an orderly series of stages is required (problem definition, design, data collection, data analysis, and results interpretation). This is the only scientific methodology that allows data to be collected directly from playing participants in competitions, without eliciting the response from the direct apprehension of perceptible information, preferably helping us through recording, which is the usual method to access information (Anguera and Mendo, 2013).

Observational methodology is a scientific procedure that allows the detection of behaviors perceiving them in their usual context, proceed with systematic recording and analysis, both qualitative and quantitative and mixed methods (Anguera et al., 2014), using a suitable instrument, enabling the detection of different types of relations and evaluating them. This will require the selection and use of the most appropriate analysis tool depending on the data collected nature (qualitative, quantitative or mixed) and the intended results (descriptive, comparative, or predictive). Observational methodology, proposes certain procedural structures –observational designs– through a set of criteria which are the natural bases of observational studies. In each study, once the objectives have been defined, the observational designs established then guide the entire process, influencing the preparation of the observation instruments, the recording and its metrics, the observational sample, data quality control and to a large extent the choice of the most appropriate analysis techniques. They also have a significant repercussion on the interpretation of the results.

In football, unlike other team sports of cooperation-opposition and simultaneous participation, due to its complex nature (Davids et al., 2005; Araújo et al., 2006; Perl, 2006), high uncertainty and multifactoriality (Gréhaigne, 2001; McGarry et al., 2002; Lames and McGarry, 2007), the search is not easy, which means that identifying factors that affect success is of particular interest (O’Donoghue, 2010). Performance in this sport has a multidimensional setting and can be grouped into two broad areas of study. On the one hand, we would find analytical factors related to conditional aspects and, on the other hand, competition factors that would require an analysis in its natural context. Within the latter, tactical-strategic aspects allow to better reflect the nature of the game and to better understand its development.

In recent years this type of work has proliferated (Hughes and Franks, 2005; Lago, 2009) aiming to detect successful play patterns through the analysis of different game situations and different variables. Some of these studies focus their interest in studying the offensive phase (Ensum et al., 2000; James et al., 2004; Jones et al., 2004; Hughes and Franks, 2005; Lago and Martín, 2007; Acar et al., 2009; Lago-Ballesteros et al., 2012; Collet, 2013; Casal et al., 2015; Ric et al., 2016), others the defensive phase (Barreira et al., 2013; Vogelbein et al., 2014; Andujar, 2015; Mohammad et al., 2016; Ric et al., 2016) and others in the analysis of situational variables (Borrás and Sainz de Baranda, 2005; Tucker et al., 2005; Taylor et al., 2008; Lago, 2009, 2012; Lago-Peñas and Dellal, 2010; Lago-Peñas and Lago-Ballesteros, 2011; De Oliveira, 2012; Sainz de Baranda and López-Riquelme, 2012; Sánchez Flores et al., 2012; Ardá et al., 2014; Casal et al., 2014, 2015).

Another element of great interest in football’s performance analysis is the identifying and understanding differences between game patterns developed by successful and unsuccessful teams (Hughes and Bartlett, 2002). To acquire objective information that permits assessing team performance (Carling et al., 2005) and differentiate playstyle from successful teams and unsuccessful ones (Mckenzie and Cushion, 2012).

One of the most studied indicators in football research has been possession (Bate, 1988; Dawson et al., 2000; Garganta, 2000; Hadley et al., 2000; Carmichael et al., 2001; Hughes and Bartlett, 2002; Hughes, 2003; McGarry and Franks, 2003). This is because it can lead a team to take the initiative of the offensive game, though it doesn’t necessarily mean to win the match. In recent years this variable has acquired greater significance due to the success of teams like F.C. Barcelona and the Spanish national team who have maintained hegemony in European and world football using a playstyle based on possession and taking the lead through keeping the ball.

This fact is reinforced by some studies that claim that greater possession implies greater team success. Hook and Hughes (2001) reported that successful teams in the UEFA Champions League, FIFA World Cup, and UEFA Euro achieved longer possession time than the unsuccessful teams. Bloomfield et al. (2005a) showed that the top three teams in the 2003–2004 English Premier League (Arsenal, Chelsea, and Manchester United) achieved longer possession time than their opponents. James et al. (2004) detected significant differences in possession between successful and unsuccessful teams from the English Premier League. Carling et al. (2005) obtained the same results in a study from the same league but in the 1996–1997 season. Grant et al. (1999) analyzed the 1998 FIFA World Cup and Hook and Hughes (2001) 2000 UEFA Champions League, both studies reaching the same conclusion, that possession is linked to team success. Casal et al. (2015) analyzed the 2008 UEFA Euro, concluding that a longer offensive phase predicts greater success, and studies Grant et al. (1999), Hook and Hughes (2001), James et al. (2004), Bloomfield et al. (2005b), Carling et al. (2005), Hughes and Franks (2005), Collet (2013), Casal et al. (2015) also corroborate the relationship between greater possession and team success.

But it seems presumptuous to claim that longer possession time ensures greater success, as the results of different studies are inconclusive and reality shows how teams with low possession time are also successful, as demonstrated by studies like those of Bate (1988) which indicates that teams are more likely to achieve goals having the ball near the goal zone and not the longer you keep the ball on your own possession, even though these two variable are often related. Stanhope (2001) also indicates that possession did not represent the successful teams of the 1994 FIFA World Cup, although it seems that the game strategies used by the successful teams have evolved over the years into a more possession based playstyle. Studies Lago and Martín (2007), Lago (2009), and Lago-Peñas and Dellal (2010) indicate that in the Spanish League greater possession is a feature observed in teams that are either losing or tying the game. Collet (2013) concludes that the effect of possession time in matches of the domestic league was negative, in the UEFA Champions League had no effect and in National team tournaments was not significant, leading to think that the influence of possession on success will depend on team capacity. Moreover, we must emphasize that in season (2015/16), according to data collected on FIFA’s official website, the top teams in the major European leagues (Bundesliga in Germany, France Ligue 1 in France, Spanish Liga, A Series in Italy and the Premier League in England) have possession times over 50% with the exception of Leicester, leader of the Premier League, which has 42% of possession during matches. Possession time or offensive phase duration could also be explained by the playstyle selected or some situational variables. Some studies have shown that possession is influenced by the match status (Sasaki et al., 1999; James et al., 2004; Jones et al., 2004; Bloomfield et al., 2005a; Lago and Martín, 2007; Taylor et al., 2008). Studies Lago and Martín (2007) and Lago (2009) found that losing teams had longer possession times in the offensive zone rather than the defensive zone.

Another variable that modulates possession time is the match location, and some studies show that home teams have longer possession times than away teams (James et al., 2002, 2004; Jones et al., 2004; Lago-Peñas and Dellal, 2010). The quality of rival team also varies possession time, being greater when facing rivals with low capacity level (Jones et al., 2004; Bloomfield et al., 2005a; Tucker et al., 2005; Lago and Martín, 2007; Lago, 2009). A transcendental aspect when possession is analyzed as a performance indicator is to discern the quality of it, as Collet (2013) advises. It will therefore be important to not only quantify the time a team retains possession during the offensive phase, but also to identify the zone in which it is carried out as keeping the ball in fruitless offensive zones (away from the goal) might not guarantee the success of the offensive phase, although it may be a recommended strategy to defend possession in circumstances that reccomend it. In this study, an analysis of ball possession of the 2016 UEFA Euro France was realized, the main objective being to identify the possible relationship between possession time and the zone in which it develops with team success, reflected in the results of the match. That is, we want to know whether the successful and unsuccessful teams are characterized by more or less possession in certain zones of the field, showing a different offensive game. The main contribution that this study provides to the scientific field is conducting a quantitative and qualitative analysis of ball possession, as it not only means to quantify the time of team possession but also to identify the area where this occurs in order to determine the quality of the same. On the other hand, performing a multivariate analysis to identify the influence of possession time and area on the outcome of the match, and identifying a model that enables us to predict team success based on these variables.

The hypothesis of this study is that team level modulates the type of ball possession, both quantitatively (possession time) and qualitatively (area of possession).

## Materials and Methods

### Participants

To control some of the situational variables that can potentially affect tactical and strategic team behavior, such as quality or level of opposing teams and the match location (Kormelink and Seeverens, 1999; Carling et al., 2005), 12 matches corresponding to the round of eighth-finals, quarterfinals, semifinals and final of the 2016 UEFA Euro France have been selected in which 2.284 ball possessions occured. Switzerland, Poland, Croatia, Portugal, Wales, Northern Ireland, Hungary, Belgium, Germany, Slovakia, Italy, Spain, France, Eire, England, and Iceland were the teams analyzed. Three games (Switzerland vs. Poland; Poland vs. Portugal and Germany vs. Italy) have been excluded from the analysis since the match outcome was a draw having in account regular time and extensions, which makes impossible to label the teams as successful or unsuccessful. This sample ensures that all matches are played on neutral ground, the teams have a similar level and, by eliminating the games of the group phase, we also make sure that the teams look for the victory in their matches, since defeat will mean elimination. In the group phase matches, it may happen that some team is more interested in drawing or losing any of their matches, to avoid a particular opponent in the following phases, this would lead to incorrect results in the study.

### Instruments

Four national coaches and experts in football research designed an ad hoc observation instrument combining a field format and category system (Anguera and Mendo, 2013) was created (**Table 1**). Variables designed for the study are time (time that teams have ball possession in each field zone, in minutes); possession zone (spatial division of the field in defensive half and offensive half); match outcome (determined based on the number of goals scored and conceded at the end of the match); match status (match result at the time of registering each possession); match half; move outcome.

**Table 1:** Category system used in the observation tool.

Criterion	Categories
Time	Possession time in each zone
Possession zone	Middle defensive zone
	Middle offensive zone
Match outcome	Win
	Draw
	Loss
Match status	Winning
	Drawing
	Losing
Match half	First half
	Second half
Move outcome	Goal
	Shot
	Own corner kick
	Opponent’s corner kick
	Own throw-in
	Opponent’s throw-in
	Own’s foul
	Opponent’s foul
	Lost possession

### Procedure

In order to carry out the study, a direct, non-participatory, systematic, and natural observational methodology was used (Anguera et al., 2011).

Matches were recorded from TV emitted images and were registered and analyzed post-event. Because the video recordings were public, confidentiality was not an issue and authorization was not required from the players observed or their representatives. Furthermore, the information cannot be considered either personal or intimate, as the research consisted solely of naturalistic observations in public places, and it was not anticipated that the recordings would be used in a manner that could cause personal harm (The American Psychological Association’s [APA’s], 2010). No experimental analysis involving human studies is performed in the study.

#### Basic Concepts

Basic concepts used in this study are, firstly, the definition of ball possession. We have adopted the definitions of two previous studies (Castellano, 2000; Casal, 2011), determined that a team starts a possession, while it is in play or when a player gets the ball while it is in possession of the other team must meet at least one of the following criteria:

(1) The player who receives the ball must touch it at least two times.
(2) The player intercepts the ball and a partner continues possession.

If the ball is stationary, a team starts a possession, when the ball has been put into play after a reglementary interruption had been decreed and consequently the match stopped. The analysis unit was composed for the entire offensive phase of the team, since ball possession started until it was lost or the match was interrupted.

Space arrangement used harnesses the subdivision performed by field regulation, dividing the field into two parts by a vertical line (central line). The zone of the field comprised between the central line and the bottom line of the goal of a team has been called middle defensive zone and the other half, bounded by the central line to the bottom line of the opposing goal has been called middle offensive zone.

Criteria used for the division of the teams into two groups, successful and unsuccessful, has been the outcome of the match (Lago-Peñas et al., 2010), excluding penalties. This way, all the teams that won their matches during reglementary time or extensions were classified as successful and teams who lost their matches as unsuccessful.

#### Data Quality Control

To try to ensure data reliability, all matches were registered and analyzed by four observers, all of them national soccer coaches with more than 10 years of experience in the field of training, teaching, and research in football through observational methodology. In addition, the following training process was carried out: First, eight observing sessions were conducted on teaching the observers following the Losada and Manolov (2014) criteria and applying the criterion of consensual agreement (Anguera, 1990) among observers, so that recording was only done when agreement was produced. To ensure inter-reliability consistency of the data (Berk, 1979; Mitchell, 1979) the Kappa coefficient was calculated for each criterion (**Table 2**), it revealed a strong agreement between observers, which means high reliability, taking Fleiss (1981) as a reference, who establishes a classification for the Kappa values where it characterizes as regular values found between 0.40 and 0.60, good between 0.60 to 0.75, and excellent above 0.75. Moreover, the procedure was repeated after 2 weeks (to exclude any learning effects) to check intraobserver reliability (Mitchell, 1979).

**Table 2:** Observers inter-reliability by criterion.

Criteria	Ob_1_-Ob_2_	Ob_1_-Ob_3_	Ob_1_-Ob_4_	Ob_2_-Ob_3_	Ob_2_-Ob_4_	Ob_3_-Ob_4_
Time	0.9	1	0.89	0.95	1	0.92
Possession zone	1	0.93	1	0.98	0.97	1
Match outcome	1	1	1	1	1	1
Match status	1	1	1	1	1	1
Match half	1	1	1	1	1	1
Move outcome	1	1	1	1	1	1
*K*_total_	0.98	0.99	0.98	0.98	0.99	0.98

#### Statistical Analysis

Variables analyzed were Match Status, Half Match, Possession Zone, Move Outcome in relation to Possession Time. In the case of possession time and match status result was significant, and was complemented by a Kruskal–Wallis *post hoc* test to know among which categories the differences existed. Half match proved to be non-significant while possession zone has a significant result.

In the case of possession time and move outcome, several play options are analyzed, applying the Kruskal–Wallis test to see if differences were found between them (Tenga and Sigmundstad, 2011).

A comparative analysis of possession zone between successful and unsuccessful teams (match outcome) was also carried out, with significant differences between both groups of teams. The size effect was calculated in terms of Cramer’s and Chupov that showed low intensity between the two variables. We also found differences between possession time and successful and unsuccessful teams, using Welch’s T. To know the size of the effect, a point-biserial correlation was applied (Nakagawa and Cuthill, 2007), indicating that a relationship exists, but with a low intensity.

Finally, a logistic regression model was performed, to know the influence that possession time and possession zone (predictor variables) have on match outcome (variable explained). The model’s degree of adjustment was verified (Ato and López, 1996; Hair et al., 1999), and once verified the success probability estimation was calculated, depending on the values of predictor variables.

To perform statistical analysis the R program (v.3.2.0) was used, libraries used were epiDisplay, pscl, BaylorEdPsych, and Modeva. Significance level for each performance indicator was set at 5%, as usual in comparable scientific studies (Taylor et al., 2005).

## Results

Agreeing with Allen (2003) definition, the most common way of describing a set of interrelated data is to calculate the mean value and a dispersion measure around this mean value. We started presenting the related values between “match status” and “possession time,” which shows that the average possession time in a winning team is 20.3 m with a deviation of ±16.0 m (*N* = 667) during the match. In case of a draw, shows a mean value of 18.2 m with a standard deviation of ±16.8 m (*N* = 912). Finally, in the case of losing the mean length of possession is 13.7 m, with a deviation of ±12.3 m (*N* = 705). The relationship between the three categories of the variable “match status” indicate that there are significant differences between them (*p*-overall < 0.01) (**Table 3**). The standard error is important, because records have a large dispersion, most are outliers.

**Table 3:** Relationship of possession time and match status.

	Winning	Drawing	Losing	*p*_overall
	*N* = 667	*N* = 912	*N* = 705	
Possession time	20.3 ± 16.0	18.2 ± 16.8	13.7 ± 12.3	<0.01

**FIGURE 1:**
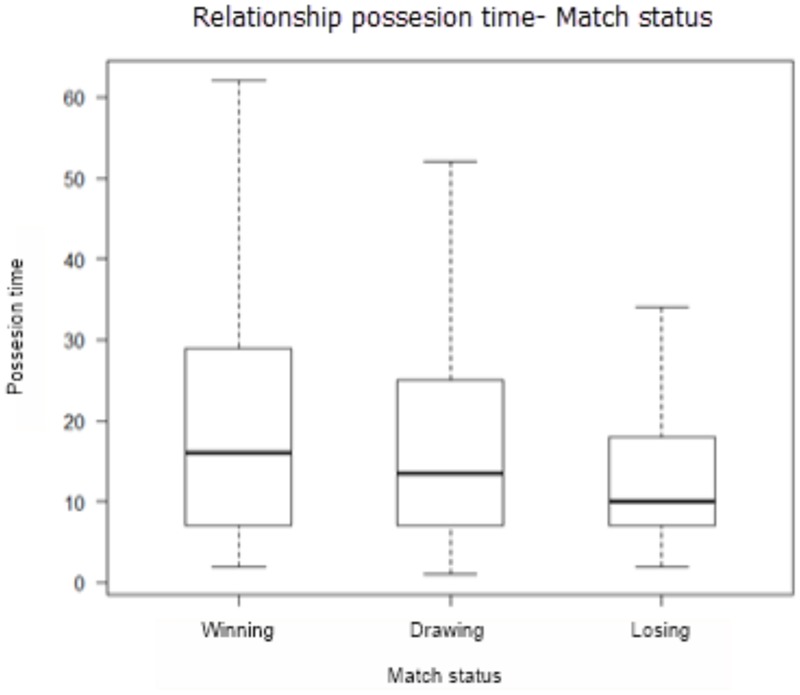
Match status and possession time diagram box.

Average “possession time” (**Figure 1**) is smaller with the result “losing.” For a “draw” result the average increases slightly, and finally presents the greatest value for the result “winning.”

In order to know among which categories the differences occur, comparisons are proposed two to two, with a Kruskal–Wallis *post hoc* test. The Kruskal–Wallis test shows a chi-square statistic value of 92,628, with *p*-value = 0.011, indicating differences between categories. In the *post hoc* contrast, significant differences are found in the WINNING–LOSING and DRAWING–LOSING pairs (**Table 4**).

**Table 4:** Categories differences based on pairwise comparisons.

	Obs.dif	Critical.dif	Difference
Winning–Drawing	26.02823	40.77737	False
Winning–Losing	75.40521	43.22467	True
Drawing–Losing	49.37698	40.12126	True

Analyzing the relationship between the variables “match half” and “possession time,” we obtained a mean value of 17.6 m with a standard deviation of ±15.3 m (*N* = 1,190 plays) in the first half, while in the second half the average length of possession is 17.2 m with a deviation of ±15.8 m (*N* = 1,094). These differences were not statistically significant (*p* = 0.73) (**Table 5**).

**Table 5:** Relationship possession time – half match.

	First half	Second half	*p*_overall
	*N* = 1190	*N* = 1094	
Possession time	17.6 m ± 15.3 m	17.2 m ± 15.8 m	0.73

**FIGURE 2:**
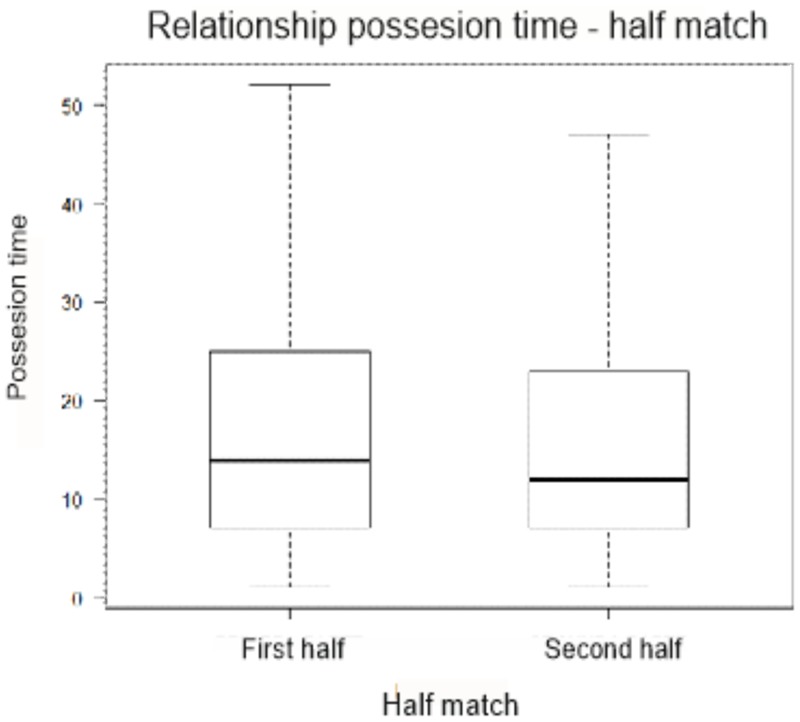
Half match and possession time diagram box.

In **Figure 2** a slight reduction in possession time is observed in the second half of the match.

Variables “possession zone” and “possession time” have a mean value of 16.0 m, with a standard deviation of 13.5 m (*N* = 1,053) in the middle defensive zone. The middle offensive zone has a mean value of 18.6 m with a standard deviation of ±17.0 m (*N* = 1,231). Differences are significant (*p* = 0.04-overall), (**Table 6**).

**Table 6:** Descriptive possession time – possession zone.

	Middle defensive	Middle offensive	
	*N* = 1.053	*N* = 1.231	*p*_overall
Possession time	16.0 m ± 13.5 m	18.6 m ± 17.0 m	0.04

**FIGURE 3:**
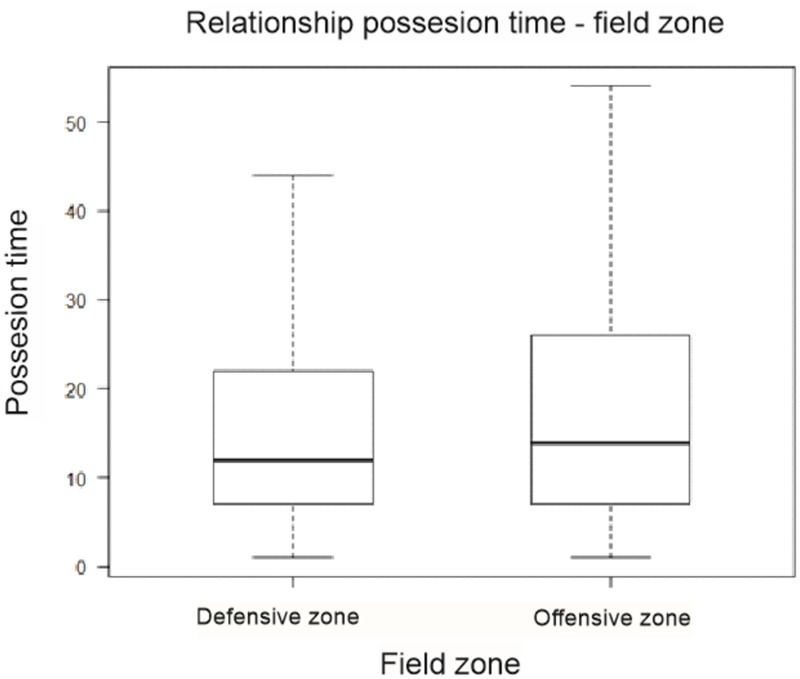
Possession zone (field zone) and possession time diagram box.

It is seen in mean values of “possession time” which is slightly lower for the middle defensive zone compared to the offensive zone. We observed that distance in the middle offensive zone in the interquartile range is greater, plus a greater dispersion of the observations (**Figure 3**). This means that the hold time is increased in this area.

**FIGURE 4:**
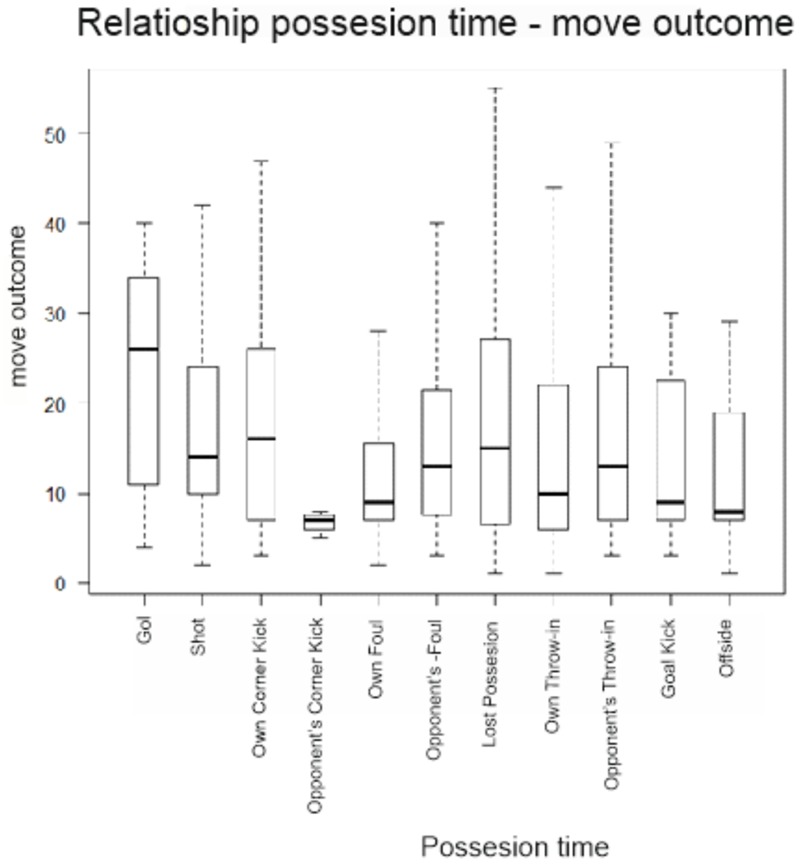
Move outcome and possession time diagram box.

In the box diagram (**Figure 4**), like the previous box diagrams (**Figure 3**), outliers have been deleted on their behalf to have a better view of the distributions of each category of move outcome based on possession time. There are differences in interquartile ranges, as well as the last values of the upper whiskers, while the difference between the values of the initial whiskers are not significant. This indicates that distributions are biased.

To find significant differences between these variables the Kruskal–Wallis test was applied, with a value of 68.062, and *p*-value = 0.3408 all means being equal.

Possession time is a success indicator identified in several works, the objective is to determine possession time and field zone in which possession occurs since having possession in the middle defensive zone does not necessarily mean more success since the ball is away from the rival goal.

**Table 7** provides an overview of use frequency of the various zones of the field and mean durations of possession in different zones, depending on successful and unsuccessful teams.

**Table 7:** Relationship between group, zone, and possession time.

Group	Field zone	Fr.	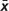 PT^∗^
**Successful**	Middle defensive	406	15.82
	Middle offensive	712	20.23
**Unsuccessful**	Middle defensive	578	14.23
	Middle offensive	588	12.74

*^∗^Possession time.*

Possession zone changes significantly between groups, *x*^2^ = 15.72, *p* < 0.05. Specifically, successful teams occupied more frequently the middle offensive zone than the unsuccessful (712 times against 588, respectively). On the other hand, the unsuccessful teams occupied a greater number of times the middle defensive zone than the successful teams (578 vs. 406).

Observation indicates that successful teams spend more time in the middle offensive zone, and is accompanied by a greater possession time (20.23 s) as contrary to the middle defensive zone (15.82 s). While unsuccessful teams spend more time inside the middle defensive zone, with a longer possession time (14.23 s) than in the middle offensive zone (12.74 s).

Intensity determined by association coefficients Cramer’s V 0.13, and Chuprov coefficient T^2^ 0.13 used to measure symmetrical association between variables showed low intensity relationship between variables.

Significant differences were also found in possession time between successful and unsuccessful teams, *H* = 24.289, *p* < 0.001. To study the relationship between possession time and match outcome Welch’s T was used.

In this case, statistic *t* = 5.408, *p* < 0.001 with a confidence interval of 95% between -7.072 and -3.305, and 18.67 in successful and 13.48 in unsuccessful teams.

**FIGURE 5:**
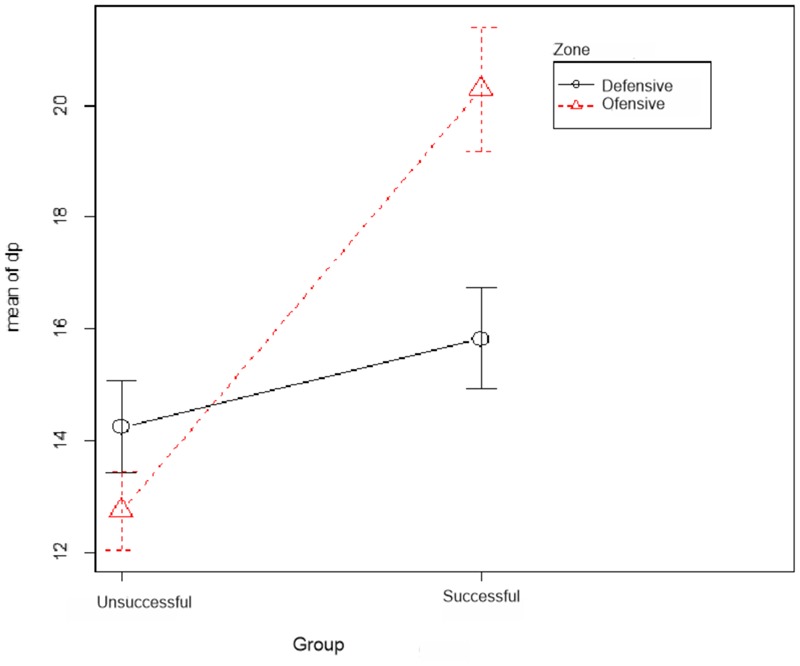
Mean comparison.

Observing the three variables (**Figure 5**) shows that in the successful teams attack patterns, teams stay longer in the middle offensive zone with a longer possession time than unsuccessful teams, while unsuccessful teams stay longer in the middle defensive zone with longer times in possession.

Size effect was measured applying a r_bp_, formula quoted by Nakagawa and Cuthill (2007) with a value of 0.32. Positive coefficient indicates that high scores on possession time implies greater success for the team, although with small intensity.

To determine the influence of possession time and possession zone in successful and unsuccessful teams a logistic regression model was used (**Table 8**).

**Table 8:** Logistic regression model.

	Estimate	*SE*	*Z*	Pr > |*z*|	Crude OR (95% CI)	Adj OR (95% CI)
(Intercept)	-0.77	0.13	5.74	9.19e - 09^∗∗∗^		
[T.Offensive]	0.51	0.13	3.67	0.01^∗∗∗^	1.72 (1.31, 2.25)	1.67 (1.27, 2.19)
Dp	0.03	0.01	5.00	5.73e - 07 ^∗∗^	1.03 (1.02, 1.04)	1.03 (1.02, 1.04)

*^∗∗^*p* < 0.05; ^∗∗∗^*p* < 0.001.*

Successful/unsuccessful=possession zone+possession time

Team success increases 1.72 times when playing on the middle offensive zone against the middle defensive zone in the one variable model. The two variable model shows an increase of 1.67. Possession time didn’t show significant differences between successful and unsuccessful teams.

Probability of being successful in explanatory variable terms, with *X*_1_ being field zone and *X*_2_ possession time is:

(1) $$P(Exitosos)=\frac{\exp^{\left(\alpha_{0}+\;\alpha_{1}X_{1}+\alpha_{2}X_{2}\right)}}{1+\exp^{\left(\alpha_{0}+\alpha_{1}X_{1}+\alpha_{2}X_{2}\right)}}=0.4425$$

44.25% is the probability of a team being successful.

**FIGURE 6:**
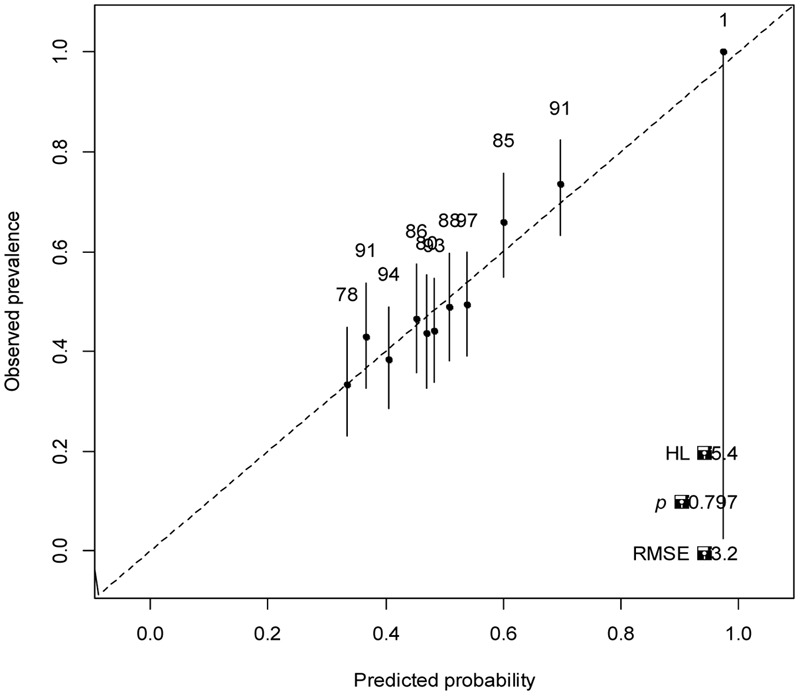
Goodness fit model.

Some authors (Hair et al., 1999) recommend using several methods to evaluate the model’s goodness of fit. A value of Hosmer–Lemeshow 0.797 indicates goodness of fit (**Figure 6**).

Evaluation model based on pseudo-coefficients *R*^2^, CoxSnell = 0.048, adjusted Nagelkerke = 0.065, Adj. McFadden = 0.036 and Tjur = 0, indicate low prediction. In classification terms, the model has 47.90% of sensitivity and 69.91% of specificity. UAC = 0.62 value with ratio of 1.23 shows an average ability to classify.

## Discussion

The main objective of this study was to determine whether possession time and possession zone are performance indicators that distinguish the successful football elite teams from the unsuccessful. What differentiates this study from its precedents is that it has tried to control some of the situational variables identified by previous studies as an influence to ball possession. Specifically, all analyzed matches were played at neutral grounds and team level was similar having into account that the teams were the best European national teams. The match status, the other situational variable identified as influential in possession time, was also analyzed to observe level of influence. In order to generalize results we have not studied only one team but analyzed several national teams in the same competition.

Study results have allowed us to detect significant differences between possession time and match status. Longest possession time occurs when teams are winning, these results are similar to those reported by Bloomfield et al. (2005b) and Taylor et al. (2008) and contradict those found by Jones et al. (2004), Lago and Martín (2007), Lago (2009), and Lago-Peñas and Dellal (2010) who indicate that teams losing or drawing have longer periods of possession. Multiple factors may explain these differences, such as playstyles adopted by the teams during competitions, since behavior may be different depending on whether it is a national competition or an international tournament, with different national teams. It has been shown that the main differences are found between the result of winning–losing and losing–drawing, while possession time does not change when teams are either winning or drawing. These findings indicate a team tendency to not change either their playstyle nor their game pattern according to the match status, using the same strategy despite the score, and teams characterized by an attack pattern of long possessions shall not change to make counterattacks or direct attacks when they get ahead on the scoreboard, but try to keep the lead through ball possession, and teams with short attacks will not change to make long attacks when changing a favorable marker to an adverse one. However, to consolidate these data, it would be interesting that future studies considered not only the encounter’s partial result, but also score difference, since possibly a team that is losing by the difference of two or more goals and has little time left for the end of the match, regardless of their style of play, will make short possessions to try to increase the frequency of finalizations. On the other hand, teams that are winning by the difference of two or more goals, lacking a short time to the end of the encounter, although their style of offensive game is characterized by short possessions, surely will try to increase the possession time not allowing the opposing team to create chances of finalization.

Results also suggest that possession time is slightly higher in the first part of the match. This can be explained due to the fact that in the second half of the match there is a greater accumulation of fatigue and therefore the player will have a lower technical and tactical level, which will cause a greater number of errors in the technical executions and the tactical decisions, consequently producing a greater number of ball losses and possession changes. The offensive game actions ending with a goal or shot are those with a longer possession time. Data is consistent with results found in the study of Casal et al. (2015) who suggest that long possessions offer a greater chance of successful outcomes. Regarding the area of the field, results show that most of the time possessions are located in the middle offensive zone, this data is consistent with results obtained by Collet (2013) which indicated the need for effective possessions, meaning these possessions should be located in dangerous places for the opponent’s team, for example, near the opponent’s goal. Effectively, for possession to be effective, it must occur as close as possible to the opposing goal, trying to disrupt the opposing team’s defense and create a chance of finalization. Ball possession happening far from the rival team’s goal and without intention to progress is totally ineffective.

Having into account team quality the bivariate analysis has allowed to draw several evidence on the possession type of different teams. Specifically, we detected significant differences in spatial occupation frequency that teams perform, successful teams occupy a greater number of times the middle offensive zone and for longer times, on the contrary, unsuccessful teams occupy most often the middle defensive zone. Data is consistent with results obtained by Bate (1988) indicating that probability of scoring a goal depends on the number of times a team gets close to the opposite goal having possession, this being an indicator of successful teams. It seems obvious that the most advantageous possession zones are those close to the goal zones of the opposing team and that maintenance of possession in zones far from the goal don’t guarantee offensive success.

Results reinforce the established by various studies (Andujar, 2015; Casal et al., 2015) showing that modern playstyle has evolved into a positional game in which possession is the fundamental argument in collective game. Playstyle has changed from a model that produced success, identified by shooting to the opposite goal, thanks to the turnovers in the middle offensive zone and short possessions, to one in which once possession has started the attacking phase becomes elaborate and parsimonious.

Significant differences were found in possession time between different group teams. Successful teams has longer possession times than unsuccessful teams. Results agree with other studies (Jones et al., 2004; Bloomfield et al., 2005a; Hughes and Franks, 2005; Lago and Martín, 2007; Lago-Peñas and Dellal, 2010) indicating that greater possession characterizes successful teams. This fact is reflected in the playstyle the best teams of both European domestic leagues and national teams, both European and worldwide are using today. The F.C. Barcelona (2015–2016 Spanish Liga and 2015 UEFA Champions League champions), Spain’s national team (2008 and 2012 UEFA Euro 2010 FIFA World Cup champions) and German’s national team (2014 FIFA World Cup champions) are characterized by an offensive playstyle that consists of taking the lead through possession, using as an overall tactical offensive model, the combination attack.

Multivariate analysis tried to describe the relationship between possession time in each zone with team successfulness. Results showed that successful teams differ significantly from unsuccessful teams in this regard. Specifically, successful teams occupied more frequently the middle offensive zone and remained longer in the same keeping possession. On the contrary, unsuccessful teams occupied more times and middle defensive zone staying longer times than successful teams. Results reinforce those obtained with bivariate analysis which agreed with the importance of being near the goal and having long possessions to ensure team success.

Logistic regression analysis allowed us to determine possession time and zone influence on the outcome of the match, as well as identifying a model that allows us to predict team success in terms of these variables. This model indicates that each time that a possession is carried out in the middle offensive zone, the chances of winning will increase 1.72 times and the probability of success having longer possession times on the middle offensive zone will be 44.25%. These data are the main potentiality of the present work since no previous investigations have been found that carry out this type of analysis, studying the relationship between possession time, possession zone and team’s successfulness, in order to identify a game pattern with greater success.

One limitation of the study has been that only national teams matches have been analyzed, so results cannot be extrapolated to other kinds of meetings, because as indicated by studies James et al. (2004), Tucker et al. (2005), Bloomfield et al. (2005a), Lago and Martín (2007), Lago (2009), and Collet (2013) the type of competition and, in particular, quality of the rival team, influences the type of possession that will be carried out in the meeting. On the other hand, we also believe that the fact of having as sample teams of an even competitive level (being the best in Europe) is work’s fortitude and, if significant differences were found between the teams, all of a similar competitive level, it is feasible that between different level teams differences will be even greater. We are also aware of the existence of other extraneous variables that can influence the results as the playstyle in different competitions (Rienzi et al., 2000), arbitration decisions, weather conditions or the state of the field, but it would be impossible controlling all of these variables, so this study has tried to show the influence of some of them.

Results obtained are expected to help giving more knowledge about successful offensive game models, as well as performance factors of the offensive phase, which will allow teams to optimize their training process and performance during the match. In the field of research contributions could prove useful in future studies of possessions, taking into consideration not only possession time but also the area in which it occurs and team quality.

## Conclusion

This study allows us to identify, characterize and differentiate different attack patterns between successful and unsuccessful teams, based on possession time and zone in which it occurs. Results show that significant differences between the two groups are found. Data establishes that successful teams are characterized by an offensive game pattern with greater possession and more presence in the middle offensive zone. On the other hand, unsuccessful teams have shown an offensive game pattern with lesser possession time. In addition, longer possession time in the middle offensive zone, predicts greater chance of victory in the match.

Current football’s empirical observation and analysis leads to the identification of a possession playstyle generalized commitment, it seems that coaches and teams have opted for this model, but what makes teams have higher success rates than others? Probably the answer is related to the individual effectiveness of the actors (players) in the collective framework. We can never forget that individualities build the collective game and, therefore, individual quality of the players is a key factor of performance, which will mark the collective success of the teams.

## Author Contributions

CAC developed the project, review the literature and wrote the manuscript. JLL was responsible for performed analysis, the method section and revised the content critically. RM and TA collected and analyzed the data and supervised the drafting of the manuscript. FJM translated the manuscript. All authors approved the final, submitted version of the manuscript.

## Conflict of Interest Statement

The authors declare that the research was conducted in the absence of any commercial or financial relationships that could be construed as a potential conflict of interest. The reviewer EP and handling Editor declared their shared affiliation, and the handling Editor states that the process nevertheless met the standards of a fair and objective review.
